# Morphokinetic parameters as auxiliary criteria for selection of blastocysts cultivated in a time-lapse monitoring system

**DOI:** 10.5935/1518-0557.20200035

**Published:** 2020

**Authors:** José Fernando de Macedo, Luiz Mauro Oliveira Gomes, Maristela Rodrigues Oliveira, Gustavo Capinzaiki Macedo, Giovanna Capinzaiki Macedo, Daniela Oliveira Gomes, Camila Dutra Souza Francisquini, Bruna Oliveira Ambrogi, Sandra Irene Sprogis dos Santos

**Affiliations:** 1 Clínica Reproferty, São José dos Campos, SP, Brazil; 2 UNIFUNVIC- Centro Universitário, Pindamonhangaba, SP, Brazil

**Keywords:** blastocyst, time-lapse monitoring system, blastocyst quality

## Abstract

**Objective:**

To describe embryonic profile up to blastocyst stage in a time-lapse system.

**Methods:**

A retrospective, longitudinal, analytical study of patients submitted to *in vitro* fertilization. The embryos were grouped according to the degree of expansion, internal cell mass and trophectoderm classification, the morphokinetic parameters were associated with the time periods stated in each evolution phase.

**Results:**

The appearance of a second polar corpuscle (CPap) occurred earlier in the embryos classified as excellent (2.99h; *p*<0.05), in relation to the embryos classified as good (3.40h), average (3.48h) and poor (3.55h). The embryos classified as excellent took less time for the pronuclei to disappear (PNbd) (21.80h; *p*<0.05), when compared to the good embryos (22.96h), the average (23.21h) and the poor (23.47h). As for the morphokinetic parameter, the end of the two-cell division (T2) occurred first in the excellent blastocysts (24.38h; *p*<0.05), when compared to the other groups: good (25.57h), average (25.53h) and poor (25.78h). With respect to synchronization with the division of three to four cells (S2), the poor embryos presented longer times for such division to occur (3.67h; *p*<0.05). When compared to the embryos from the groups excellent (1.97h), good (2.70h) and average (2.09h). At the time point of the blastocoel formation (TB), the excellent embryos (104.04h) did not differ from the good embryos (104.10h). However, when compared to average (107.27h) and poor (106.86h) embryos, there was statistical significance (*p*<0.05).

**Conclusions:**

Embryos of better quality had a shorter time in some morphokinetic parameters when compared to the other groups, thus increasing the possibilities to establish new parameters for the classification and selection of embryos.

## INTRODUCTION

Despite efforts to improve outcomes in assisted reproduction processes, pregnancy rates are relatively low, and in order to increase these percentages the transfer of multiple embryos per cycle has been performed, which can lead to an increase in multiple pregnancies, which is associated with neonatal complications and maternal health problems (Svendsen *et al*., 1996; Alasmari *et al*., 2016; Klitzman, 2016; Wintner *et al*., 2017).

In recent years, efforts to improve embryo selection in assisted reproduction cycles have been directed to laboratory practice. There is a documented correlation between the morphological characteristics and the stage of embryo development at certain times and its parameters. In order to reduce the number of embryos in the transfer without reducing the chances of pregnancy, we have been seeking the best way to identify high implantation capacity embryos (Meseguer *et al*., 2011; Rubio *et al*., 2014; Macer *et al*., 2017). One of the non-invasive and objective evaluation methods to distinguish embryos has been the Time-Lapse (MTL) monitoring system with the use of EmbryoScope (Vitrolife A/S, Denmark^?^). This tool enables the assessment of morphokinetic parameters in order to select embryos with greater implantation potential, and minimize human handling (Marcos *et al*., 2015; Peavey *et al*., 2015; Bodri *et al*., 2016).

Recent studies have suggested that this monitoring can introduce dynamic markers of embryonic competence as well as offer the possibility of monitoring for 24 hours during embryo development. In this way, there is an increase in the quantity and quality of information, without disturbing the conditions of the culture medium, since the embryos remain in a stable and controlled environment (Kirkegaard *et al*., 2013a; Tejera *et al*., 2013; Drejza *et al*., 2017).

The main objective of this study was to provide information regarding parameters that could be relevant, and thus guide on the choice of the best embryo for transfer during the *in vitro* fertilization process. The specific objectives were: - to classify the blastocysts as to excellent, good, average and poor, so as to achieve the best embryo transfer profile to use in IVF. Trace the embryo development profile during 24 hours until expanded blastocyst, in groups of embryos classified as Excellent, Good, Average and Poor; Compare the results obtained among the studied groups by analyzing how these factors interfere in the final quality of these embryos according to Gardner and Lane’s classification (Schoolcraft *et al*., 1999).

Thus, we expect to shed light on the most suitable profile for a possible clinical indication of the best embryos to be transferred or frozen for *in vitro* fertilization processes.

## MATERIALS AND METHODS

This is a retrospective, longitudinal, analytical study; involving patients who underwent an IVF procedure in the Assisted Reproduction program of a clinic in the Metropolitan Region of Vale do Paraiba/SP, from September 2018 to March 2019, which was submitted for approval from the Research Ethics Committee.

### Inclusion criteria

We evaluated all the cases in which there was an evolution from embryo to blastocyst.

### Exclusion Criteria

Those patients who were not grouped according to the study variables.

### Stimulation protocol

All women in the study were treated with GnRH antagonist, and ovarian hyperstimulation was considered with the administration of gonadotropins at doses adjusted according to clinical response. Human chorionic gonadotropin (hCG) was given to trigger ovulation when the follicles reached values greater than 18mm in diameter. Embryonic culture removal of the oocytes was performed 36 hours after hCG administration, and they were inseminated by intracytoplasmic sperm injection (ICSI) and placed in culture medium. The embryos were grown in a special incubator, EmbryoScope (Vitrolife A/S, Denmark).

### Sample

The embryos were classified according to their degree of expansion, MCI, and trophectoderm. The total number of blastocysts studied was 337, from which 136 were classified as A (Excellent); 32 were classified as B (Good); 89 were C (Average), and 80 were D (Poor).

The embryos used for the study were grouped according to quality, and divided into A (Excellent): 3AA, 4AA, 5AA and 6AA; B (Good): 3AB, 4AB and 4BA; C (Average): 3BB, 4BB, 5BB, 6BB, 3AC, 4AC and 3CA, and D (Bad): 3BC, 4BC, 5BC, 3CB, 3CC, 4CC and 5CC; according to the degree of trophectoderm cohesion; we compared the internal cell mass and degree of expansion of the blastocoel to the morphokinetic parameters (times of cell division) (Zhao *et al*., 2018).

### Morphokinetic parameters

Meseguer*et al.* (2011) parameters were defined from the time ICSI was performed, and the second polar corpuscle (CPap) was seen; pronuclei appearance (PNap); pronuclei disappearance (PNbd); end of the division into two cells (T2); end of the division into three cells (T3); end of the division into four cells (T4); end of the division into five cells (T5); duration of the second cell cycle (Cc2 / T3-T2); time between the division of three cells and five cells (Cc3 / T5-T3); synchronization of the three to four cells division (S2 / T4-T3); when the blastocyst begin to expand, the embryo grows in size, and the zona thins out (TB)

### Blastocyst Classification

The quality of blastocysts was classified according to Gardner and Lane’s (Schoolcraft *et al*., 1999) Morphological Assessment System, as well as the degree of trophectoderm cohesion, the internal cell mass, and degree of blastocoel expansion.

### Data collection

We collected the data from the GS-Doctor software (Golden Skill-it solution) and passed it to spreadsheets coded by the researcher responsible for the other members of the team, guaranteeing patient confidentiality under study, according to the ethical norms of CONEP/MS.

### Statistical evaluation

We plotted the data by medium and standard deviation. We analyzed the results using the ANOVA test to compare the groups studied (analysis of variance test). We used the Tukey's test to compare mean values using the 5 AS Program-South Western Sydney PHN software. A *p* value <0.05 was considered significant.

## RESULTS

We noticed that there were 337 embryos in the blastocyst stage; the number of excellent embryos (136) was higher than in other groups.

Table 1 shows the comparison between the groups mentioned above vis-a-vis the morphokinetic parameters obtained in a time-lapse monitoring system.

**Table 1:** Morphokinetic parameters (mean ± standard deviation) obtained from the time-lapse monitoring system of blastocyst culture, according to embryo classification groups

	Classification of degree of expansion, MCI and trophectoderm			
Morphokinetic parameters	Excellent	Good	Average	Poor
CPap (h)	2.99±0.74^a^	3.48±1.34^b^	3.40±1.36^b^	3.55±1.93^b^
PNap (h)	8.20±2.42	8.66±1.62	8.89±2.10	9.17±2.65
PNbd (h)	21.80±2.58^a^	22.96±3.03^b^	23.21±2.92^b^	23.47±3.70^b^
T2 (h)	24.38±2.54^a^	25.57±3.48^b^	25.53±3.11^b^	25.78±3.96^b^
T3 (h)	34.40±3.98	34.30±4.86	35.90±4.83	35.24±5.65
Cc2 (h)	9.46±4.79	9.91±3.67	10.10±4.01	9.50±4.55
T4 (h)	37.49±5.23	37.97±6.02	37.92±4.30	37.95±5.63
S2 (h)	1.97±3.33^a^	2.70±3.43^a^	2.09±3.58^a^	3.67±4.86^b^
T5 (h)	46.09±7.51	46.66±5.99	47.96±7.20	46.74±7.84
Cc3 (h)	11.69±5.57	11.36±1.87	11.14±9.31	11.49±5.01
TB (h)	104.04±8.53^a^	104.10±10.72^b,a^	107.27±8.73^b^	106.86±8.44^b^

Appearance of the second polar corpuscle (CPap); appearance of the pronuclei (PNap); disappearance of pronuclei (PNbd); end of the division into two cells (T2); end of the division into three cells (T3); duration of the second cell cycle (Cc2 / T3-T2); end of the division into four cells (T4); synchronization of the division of three to four cells (S2 / T4-T3); end of the division to five cells (T5); time between the division of three cells and five cells (Cc3 / T5-T3); when the blastocyst began to expand (TB).

a,b Values within rows the diferente superscripted letter are significantly different

Second polar corpuscle (CPap) could be seen, based on the results obtained, occurring earlier in embryos classified as excellent (2.99h; *p*<0.05), when compared to embryos classified as good (3.40h), average (3.48h), and poor (3.55h).

Embryos classified as excellent took less time to have their pronuclei disappear (PNbd) (21.80h; *p*<0.05), when compared to good (22.96h), average (23.21h), and poor (23.47h) embryos.

As for the morphokinetic parameter, the end of the two-cell division (T2) occurred first among the excellent blastocysts (24.38h; *p*<0.05) when compared to the other groups: good (25.57h), average (25.53h), and poor (25.78h). Regarding the synchronization of the three-to-four-cell (S2) division, poor embryos took longer to divide (3.67h; *p*<0.05) when compared to the excellent (1.97h), the good (2.70h), and the average ones (2.09h).

Upon assessing the onset of blastulation (TB), the excellent embryos (104.04h) did not differ in relation to the good embryos (104.10h). However, when compared to the average (107.27h) and the poor (106.86h) ones, there was statistical significance (*p*<0.05).

Regarding the parameters: appearance of the pronuclei (PNap), the end of the division to 3 cells (T3); duration of the second cell cycle (Cc2); the end of the division to 4 cells (T4); the end of the division to 5 cells (T5) and time between the division to 3 and 5 cells (Cc3), there was no significant statistical difference among the blastocysts in the studied groups.

Regarding the results, it is possible to see that better-quality embryos had lower times in some morphokinetic parameters when compared to the other groups, thus increasing the possibilities of establishing new embryo classification and selection parameters.

## DISCUSSION

The Time Lapse Embryo Monitoring System (MTL) is the most recent technology developed for evaluation and selection of embryos with high implantation capacity. This technique enables the collection of more information about the *in vitro* development of the embryos through monitoring the embryo for 24 hours. In addition, embryos are not removed from the culture environment, and the morphokinetic information and noninvasive conditions during culture are useful in selecting the most appropriate embryo (Kirkegaard *et al*., 2015; Kovacs, 2016).

In this study, we used the morphological characteristics according to Gardner and Lane’s (Schoolcraft *et al*., 1999) classification to classify the blastocysts as excellent, good, average or poor. Table 1 shows statistically significant values for the morphokinetic parameters defined as: appearance of the second polar corpuscle (CPap); disappearance of the protons (PNbd); termination of the division into two cells (T2); synchronization of the 3 to 4 cell division (S2) and time at which the blastocyst began to expand (TB).

In this sense, Meseguer *et al.* (2011) defined a hierarchical prediction model combining statistical evaluation with the dynamic parameters and the most predictive parameters of that study, according to the authors, were the times of T5, S2 and Cc2 occurrence. The S2 parameters were also defined and used in this study; and there was agreement of results for the S2 time. Already at the end of division to 5 cells (T5), and duration of the second cell cycle (Cc2), did not show statistically significant differences between the groups studied.

In relation to the PNbd, there was statistical significance for the embryos classified as excellent with the time, when compared to the good, average and poor embryos. Although the significant statistical difference between the excellent and the embryos of the other groups was revealed, all the embryos of this study, regardless of the group, showed less time than Azzarello *et al*. (2012), who proved it (24.9h) to be associated with the prediction of live births.

Coticchio *et al*. (2018), using MTL, reported that male and female pronuclei (PN) appeared simultaneously 6.2 hours after ICSI. However, the initial position of the male CP can be cortical, intermediate or central in the following proportions, respectively: 15%, 31%, 2% and 53.8%. They also revealed that PN juxtapositions involve rapid movements of the female PN towards the male PN. Thus, PN disappearance times and the first cleavage showed a consistent relationship, occurring progressively later, depending on whether the position of the initial male PN was central, intermediate or cortical. In this way the time intervals between fertilization events were strongly associated with embryo quality on day three.

In this study we found that in relation to the T2 parameter, the same occurred, where the excellent embryos showed less time when compared to the good, average and poor groups. Lee *et al*. (2012) reinforced, in consonance with other authors (Pfeffer *et al*., 2005; Fu *et al*., 2009), that early cleavage in the first embryonic division resulting in two cells, about 25 to 27 hours post ICSI is a relevant data, since there are several reports describing the transfer of embryos with cleavage. Precocious cells have high implantation rates and some studies still state that higher rates occur in the formation of blastocysts in early cleaved embryos.

In our results, the embryos classified as poor showed longer time for second generation cell division when compared to the embryos of the excellent, good, and average groups. Data by Chen *et al*. (2013) show that better quality blastocysts developed under significantly lower times for second-generation cell divisions. Our data substantially support these findings.

In addition, they further state that ICSI time for the five-cell stage and for the morula stage were indicative of good blastocyst quality. In the study by Ciray *et al*. (2014), the researchers state that the time that the embryo passes from three to five cells was confirmed as a key parameter, associating it with higher implantation rates in comparison to other evaluated criteria. However, when we evaluated the time between the division to 3 and 5 cells (Cc3), these findings were not significant.

Hashimoto *et al.* (2012) and Dal Canto *et al.* (2012) highlighted the third generation of cell division, the stages of four to eight, or five to eight cells, as indicative of expanded blastocysts. This study has not focused on evaluating the time of division to eight cells.

Regarding blastulation onset (TB), the excellent embryos did not differ in relation to the good embryos; but when compared to the average and poor embryos, there was a significant difference. Herrero *et al.* (2013) found time differences related to embryo morphology in the cleavage and blastocyst stages, highlighting the time of 104.1 hours for blasting into embryos with a high probability of implantation.

These results demonstrate clear and precise conclusions, showing the existence of a relationship between each stage of development with its predictive power for the same phase, so that the earliest parameters are those that allow predicting the initial development potential.

In addition to the focus on the definition of parameters to select the best embryos, other studies have described new criteria to be analyzed, such as Kirkegaard *et al*. (2013b) in relation to the influence of oxygen on embryo stability, corroborating the early cleavage and obtaining higher rates of term pregnancy in the development of embryos with the use of time lapse.

Other morphokinetic criteria for blastocyst development and rates of implantation and live births (Kovacs, 2016) were used in heterogeneous populations under non-standardized culture conditions, thus increasing the range of possibilities to establish new parameters for the best use of MTL.

### Limitations

More comprehensive and well-designed studies must be performed to evaluate the effectiveness of time lapse in clinical use, considering the recent clinical technology and its potential new variables, which will be introduced for better use, but future revised guidelines are required.

## CONCLUSIONS

Embrioscope is the latest technology proposed for the evaluation and selection of embryos; and through this study, data can be obtained to report on the morphokinetic profile of the population of a human reproduction clinic in the Metropolitan Region of the Paraiba Valley, in order to obtain tools to develop new selection criteria or to fine-tune existing ones for a more widespread use of Time-lapse monitoring.

We inferred the importance of establishing blastocyst parameters with greater implantation potential, a subject already extensively discussed in the literature.

The morphokinetic parameters appearance of the second polar corpuscle (CPap); disappearance of the protons (PNbd); termination of the division into two cells (T2); synchronization of the 3 to 4 cell division (S2) and the time when the blastocyst began to expand (TB), had reduced times in the group of excellent embryos. Thus, the selection of better embryos through this technique will reduce the number of embryos in the transfer, without reducing the chances of pregnancy, and in the future it will minimize the problem caused by excess frozen embryos, in order to guarantee greater possibilities of obtaining a term pregnancy.

On the other hand, MTL has been only recently introduced IVF laboratories across the world, and because it is a high-cost device, more studies are needed to prove the real cost-effectiveness in obtaining better live birth rates.

## References

[r1] Alasmari NM, Son WY, Dahan MH (2016). The effect on pregnancy and multiples of transferring 1-3 embryos in women at least 40 years old. J Assist Reprod Genet.

[r2] Azzarello A, Hoest T, Mikkelsen AL (2012). The impact of pronuclei morphology and dynamicity on live birth outcome after time-lapse culture. Hum Reprod.

[r3] Bodri D, Sugimoto T, Yao Serna J, Kawachiya S, Kato R, Matsumoto T (2016). Blastocyst collapse is not an independent predictor of reduced live birth: a time-lapse study. Fertil Steril.

[r4] Chen AA, Tan L, Suraj V, Reijo Pera R, Shen S (2013). Biomarkers identified with time-lapse imaging: discovery, validation, and practical application. Fertil Steril.

[r5] Ciray HN, Campbell A, Agerholm IE, Aguilar J, Chamayou S, Esbert M, Sayed S, Time-Lapse User Group (2014). Proposed guidelines on the nomenclature and annotation of dynamic human embryo monitoring by a time-lapse user group. Hum Reprod.

[r6] Coticchio G, Mignini Renzini M, Novara PV, Lain M, De Ponti E, Turchi D, Fadini R, Dal Canto M (2018). Focused time-lapse analysis reveals novel aspects of human fertilization and suggests new parameters of embryo viability. Hum Reprod.

[r7] Dal Canto M, Coticchio G, Mignini Renzini M, De Ponti E, Novara PV, Brambillasca F, Comi R, Fadini R (2012). Cleavage kinetics analysis of human embryos predicts development to blastocyst and implantation. Reprod Biomed Online.

[r8] Drejza MA, Kort JD, Behr B (2017). Do embryo time-lapse parameters predict euploid embryo transfer outcomes?. Fertil Steril.

[r9] Fu J, Wang XJ, Wang YW, Sun J, Gemzell-Danielsson K, Sun XX (2009). The influence of early cleavage on embryo developmental potential and IVF/ICSI outcome. J Assist Reprod Genet.

[r10] Hashimoto S, Kato N, Saeki K, Morimoto Y (2012). Selection of high-potential embryos by culture in poly(dimethylsiloxane) microwells and time-lapse imaging. Fertil Steril.

[r11] Herrero J, Tejera A, Albert C, Vidal C, de los Santos MJ, Meseguer M (2013). A time to look back: analysis of morphokinetic characteristics of human embryo development. Fertil Steril.

[r12] Kirkegaard K, Kesmodel US, Hindkjær JJ, Ingerslev HJ (2013a). Time-lapse parameters as predictors of blastocyst development and pregnancy outcome in embryos from good prognosis patients: a prospective cohort study. Hum Reprod.

[r13] Kirkegaard K, Hindkjaer JJ, Ingerslev HJ (2013b). Effect of oxygen concentration on human embryo development evaluated by time-lapse monitoring. Fertil Steril.

[r14] Kirkegaard K, Ahlström A, Ingerslev HJ, Hardarson T (2015). Choosing the best embryo by time lapse versus standard morphology. Fertil Steril.

[r15] Klitzman R (2016). Deciding how many embryos to transfer: ongoing challenges and dilemmas. Reprod Biomed Soc Online.

[r16] Kovacs P (2016). Time-lapse embryoscopy: Do we have an efficacious algorithm for embryo selection?. Reprod Biotechnol Fertil.

[r17] Lee MJ, Lee RK, Lin MH, Hwu YM (2012). Cleavage speed and implantation potential of early-cleavage embryos in IVF or ICSI cycles. J Assist Reprod Genet.

[r18] Macer ML, Barritt J, Surrey MW, Danzer H, Ghadir S, W Chang, Pisarska MD (2017). Pregnancy outcomes following single versus double euploid embryo transfer. Fertil Steril.

[r19] Marcos J, Pérez-Albalá S, Mifsud A, Molla M, Landeras J, Meseguer M (2015). Collapse of blastocysts is strongly related to lower implantation success: a time-lapse study. Hum Reprod.

[r20] Meseguer M, Herrero J, Tejera A, Hilligsøe KM, Ramsing NB, Remohí J (2011). The use of morphokinetics as a predictor of embryo implantation. Hum Reprod.

[r21] Peavey M, Kaskar K, Zarutskie P, Gibbons WE (2015). Limited clinical implementation of embryoscope during first year use at an academic institution. Fertil Steri.

[r22] Pfeffer J, Taar J, Zerah S, Labourier P, Kutner J, Raveneau P (2005). Early Cleavage Embryo a Good Quality Indicator at Embryo Transfert?. Fertil Steril.

[r23] Rubio I, Galán A, Larreategui Z, Ayerdi F, Bellver J, Herrero J, Meseguer M (2014). Clinical validation of embryo culture and selection by morphokinetic analysis: a randomized, controlled trial of the EmbryoScope. Fertil Steril.

[r24] Schoolcraft WB, Gardner DK, Lane M, Schlenker T, Hamilton F, Meldrum DR (1999). Blastocyst culture and transfer: analysis of results and parameters affecting outcome in two in vitro fertilization programs. Fertil Steril.

[r25] Svendsen TO, Jones D, Butler L, Muasher SJ (1996). The incidence of multiple gestations after in vitro fertilization is dependent on the number of embryos transferred and maternal age. Fertil Steril.

[r26] Tejera A, Herrero J, Rubio I, Castelló D, Pellicer A, Meseguer M (2013). O-210 Embryo oxygen consumption is directly related with exact timing of cell cleavages and implantation rates. Session 57: time lapse: the real revolution for ambryo assessment? Hum Reprod.

[r27] Wintner EM, Hershko-Klement A, Tzadikevitch K, Ghetler Y, Gonen O, Wintner O, Shulman A, Wiser A (2017). Does the transfer of a poor quality embryo together with a good quality embryo affect the In Vitro Fertilization (IVF) outcome?. J Ovarian Res.

[r28] Zhao YY, Yu Y, Zhang XW (2018). Overall Blastocyst Quality, Trophectoderm Grade, and Inner Cell Mass Grade Predict Pregnancy Outcome in Euploid Blastocyst Transfer Cycles. Chin Med J.

